# Placebo acceptability in chronic pain patients: More dependent on application mode and resulting condition than on individual factors

**DOI:** 10.1371/journal.pone.0206968

**Published:** 2018-11-06

**Authors:** Tilman Wolter, Barbara Kleinmann

**Affiliations:** University Hospital Freiburg, Interdisciplinary Pain Centre, Freiburg, Germany; Leiden University, NETHERLANDS

## Abstract

**Introduction:**

Placebo effects can be very effective in certain pain conditions, but their use is still highly controversial. Several studies show that patients would accept a placebo treatment under certain circumstances, particularly when they are informed prior to the treatment or when there are no effective treatment alternatives. This study examines the question, which factors influence the degree of acceptability of a hypothetical placebo application.

**Methods:**

Patients filled in a questionnaire dealing with placebo applications. Moreover general data, diagnosis, duration of pain, pain ratings and anxiety/depression/stress scores, sleep disorders and opioid intake were collected from the patients` charts. 129 patients (44 men / 85 women, mean age 51.5 years, 18.0–80.9 years) entered the study. All patients had chronic pain syndromes and were treated in an universitary academic interdisciplinary pain center. Mean duration of pain was 14.7 years.

**Results:**

The study did not show significant differences in placebo acceptability among patients with different pain diagnoses or accompanying psychological diagnoses or disorders. Hidden placebo application was considered much more unacceptable for the patients than the enhanced placebo or the open placebo application. An improved condition was associated with less feeling of deception, more trust and less negative mood than an unchanged or worsened condition.

**Conclusion:**

Acceptance of placebo as pain therapy is much more dependent on the way of application (hidden or open) or on the resulting condition (improved, unchanged or worsened) than on factors inherent in the individual patients.

## Introduction

Medical treatments, medications, injections and even operations exert their impact not only by means of biological or physiological effects. There are also psychological or functional effects such as expectations [1,2] and the bond of trust [3] between the physician and the patient. These effects usually are termed placebo effects [4,5]. Placebo effects have been widely studied in pain medicine. Placebo medication can be highly effective in certain painful conditions. Large effect sizes were found i.e. in acute pain models [6] and in irritable bowel syndrome [7]. In acute pain models, mean effect sizes of *d* = 0.81 were found when short-term stimuli were used and of *d* = 0.96 when long term stimuli were used, as a meta-analysis demonstrated [8]. Placebo effects can even exert effects as strong as surgery [9]. In a recent study, Müller et al. observed a placebo-induced reduction in chronic pain of 42% with an effect size of 1.40 in placebo responders [10]. Even open label placebos can be significantly more effective than no treatment, as studies in irritable bowel syndrome [7], migraine [11] and in chronic low back pain [12] demonstrate. Interestingly, a recent open label placebo study conducted in healthy volunteers with a heat pain paradigm, demonstrated diminished heat pain and unpleasantness ratings, when open label placebo was applied with a rationale (the explanation, that placebos can exert strong effects) [13]. In depression, an open-label study on 20 patients did not show significant of effects on the outcome, as rated in the Hamilton Scale of depression. However, medium effects size were found [14].

Conditioning plays an important role in placebo application [15,16]. In pharmacotherapy conditioning can employed in dose reducing strategies, where the pharmacological agent is intermittently replaced by a placebo. This procedure is termed Placebo-controlled dose reduction (PCDR) [17]. Open label PCDR has i.e. been described in the treatment of attention deficit hyperactivity disorder (ADHD) [18,19] but not yet in chronic pain therapy.

The clinical use of placebo is still highly controversial [20]. On the one hand, several studies show a high percentage of clinicians having at least occasionally prescribed placebos [21,22]. On the other hand, the application of a placebo might also undermine the patient’s confidence in the physician [23]. For this reason, placebo applications, at least those not previously communicated to the patient, are considered unethical and should not be carried out. The American Pain Society i.e. wrote “The deceptive use of placebos and the misinterpretation of the placebo response to discredit the patient’s pain report are unethical and should be avoided” [24].

There are some studies in the medical literature concentrating on the acceptance of placebo treatments [25–27]. These studies showed that patients would accept placebo treatment under certain circumstances, particularly if they are informed about it prior to the treatment, or if there are no effective treatment alternatives. Thus, placebo applications are not always unacceptable for the patients. In case of an open application or missing alternatives, some patients think that placebo indications are acceptable for them [27]. As even an open placebo application has in some instances demonstrated to be effective and also conditioned placebo applications have shown efficacy [17], the question of patients’ placebo acceptance becomes more important. However, the findings in the present literature on placebo acceptability are not generalizable, as they are based on only few surveys conducted with a limited number of healthy participants [25,26] or participants suffering from chronic musculoskeletal pain [27], excluding other more severe pain disorders. These studies focused more on the impact of the placebo condition on acceptability than on the impact of patient inherent factors. They found that placebo acceptability was highly dependent on the context of the intervention.

We therefore sought to broaden the present knowledge on placebo acceptability also to severely affected patients with chronic pain. Further, the present study examines the question, which factors influence the degree of acceptability of a hypothetical placebo application. In particular, we wanted to elucidate whether sex, age, pain duration, pain levels, anxiety, depression, and stress scores or different pain diagnoses, beyond musculoskeletal pain alone, have an influence on the acceptability of a hypothetical placebo application. To this end, we used a modified version of the questionnaire used by Kisaalita et al. [27] and applied it to the findings of a chart review.

## Material and methods

### Patients

All patients with chronic pain who were treated at the Interdisciplinary Pain Center of the University Hospital Freiburg, either with an interdisciplinary assessment of their pain or with multimodal pain treatment were eligible for the study. In our institution, new patients infrequently receive monodisciplinary outpatients` appointments as first contact. Mostly, patients are diagnosed during an interdisciplinary assessment. This is followed by multimodal pain therapy in many cases. Data were collected during a three-month period (5/1/2016-7/31/2016). Patients were asked to fill in a questionnaire about placebo applications. Patients signed a written declaration of consent to the study.

The study was approved by the Ethics Committee of the University Hospital Freiburg (IRB number: 121/16). The datasets generated and analysed during the current study are available as a supporting information file.

### Questionnaires

A questionnaire was distributed to the patients during their stay in the hospital. Prior to filling in the questionnaire, the patients read a fact sheet about the study and signed a declaration of agreement to participation in the study. The questionnaire contained modified questions partially derived from the questionnaire used by Kisaalita et al. [27]. The first part of the questionnaire contained questions regarding how acceptable placebo application would be for the patients under various circumstances (Table 1). The patients could answer these questions on an 11-point scale ranging from 0 (“completely acceptable”) to 10 (“completely unacceptable”).

**Table 1:** Survey questions on placebo acceptability.

Question	Short term
How acceptable would it be for you, if your physician would prescribe a placebo as a pain medication to you without telling you in advance?	Hidden placebo
How acceptable would it be for you, if your physician would tell you that he would prescribe a placebo as a pain medication to you?	Open placebo
How acceptable would it be for you, if your physician would tell you, that he would prescribe a placebo as a pain medication to you, which could enhance the efficacy of your usual pain medication?	Enhanced placebo
How acceptable would it be for you, if your physician would tell you, that he would prescribe a placebo as a pain medication to you, although other effective treatments are available?	Placebo in spite of alternatives
How acceptable would it be for you, if your physician would tell you, that he would prescribe a placebo as a pain medication to you, when no other effective treatments are available?	Placebo with no alternatives
How acceptable would it be for you, if your physician would tell you, that he would prescribe a placebo as a pain medication to you, to see if your pain was real?	Diagnostic placebo

The second part contained questions regarding the patient’s ratings scale on how deceptive placebo treatment would be for them under certain circumstances, how much they would lose trust to the doctor and how much this would negatively impact their mood (Table 2). The patients answered these questions on an 11-point scale ranging from 0 (not deceptive, no loss of trust, no negative impact on mood) to 10 (totally deceptive, complete loss of trust, total negative impact on mood). Only patients who completed the questionnaire were included in the study.

**Table 2:** Survey questions on placebo deception, trust and impacted mood.

Question	Short term
You see your doctor for pain treatment. He gives you a prescription and tells you that this medication is a powerful pain medication. After two weeks your pain has improved. You received a placebo.	Hidden placebo- improved
How deceptive would that be for you?	Deception
How much would you lose trust to this doctor?	Trust
How much would that negatively impact your mood?	Mood
You see your doctor for pain treatment. He gives you a prescription and tells you that this medication is a powerful pain medication. After two weeks your pain is unchanged. You received a placebo.	Hidden placebo- unchanged
How deceptive would that be for you?	Deception
How much would you lose trust to this doctor?	Trust
How much would that negatively impact your mood?	Mood
You see your doctor for pain treatment. He gives you a prescription and tells you that this medication is a powerful pain medication. After two weeks your pain is worsened. You received a placebo.	Hidden placebo- worsened
How deceptive would that be for you?	Deception
How much would you lose trust to this doctor?	Trust
How much would that negatively impact your mood?	Mood
You see your doctor for pain treatment. He gives you a prescription and tells you that this medication is a powerful pain medication or a placebo. After two weeks your pain has improved. You received a placebo.	Open placebo- improved
How deceptive would that be for you?	Deception
How much would you lose trust to this doctor?	Trust
How much would that negatively impact your mood?	Mood
You see your doctor for pain treatment. He gives you a prescription and tells you that this medication is a powerful pain medication or a placebo. After two weeks your pain is unchanged. You received a placebo.	Open placebo- unchanged
How deceptive would that be for you?	Deception
How much would you lose trust to this doctor?	Trust
How much would that negatively impact your mood?	Mood
You see your doctor for pain treatment. He gives you a prescription and tells you that this medication is a powerful pain medication or a placebo. After two weeks your pain is worsened. You received a placebo.	Open placebo- worsened
How deceptive would that be for you?	Deception
How much would you lose trust to this doctor?	Trust
How much would that negatively impact your mood?	Mood

### Chart reviews

General data, diagnosis based on the ICD (International Classification of Diseases), duration of pain, the presence of a sleep disorder and opioid intake were derived from the patients’ charts. Patients at our institution routinely fill in the German pain questionnaire [28] prior to admission. From this questionnaire, which is filed in the charts, pain ratings on the 11-point numerical rating (NRS) scale were taken. Patients rated the average and the highest pain intensity during the preceeding four weeks and the pain intensity they would consider tolerable. Furthermore, anxiety, depression and stress scores as measured by the German version [29] of the Depression Anxiety Stress Scale (DASS) [30,31] were taken from the charts. This scale consists of 21 questions. Seven question each refer to depression, anxiety and stress. In each question maximally three points can be scored, equalling a maximal score of 21 in each category. Cut off values are: 10 for depression, 6 for anxiety and 10 for stress [29]. Somatic and psychological diagnoses were taken from the patients`charts. Somatic diagnoses were further grouped according body region into the following categories: headache and facial pain, neck pain, low back pain, neuropathic pain and widespread pain. Psychological diagnoses were grouped into the following categories: chronic pain disorder (with somatic and psychological factors, ICD-10: F45.41), depression (mild or medium), anxiety, psychosocial factors, sleep disorder. Opioids were grouped to strong opioids (morphine, hydromorphone, oxycodone, oxymorphone, and tapentadol) and weak opioids (tramadol, tilidine) [32].

### Statistical analysis

A computer software package (GraphPad Prism, Version 5.01, GraphPad Software, Inc. La Jolla, USA) was used to conduct statistical analyses. Initially, descriptive statistics were applied to all measures. Data were tested for normal distribution by means of the D’Agostino-Pearson normality test. The Wilcoxon signed rank test was used to compare ranks in measures without normal distribution. The Mann Whitney test was used to calculate sex differences in placebo acceptability. A Friedman test with Dunn`s Multiple Comparison test was used to calculate the variance of ranks among the scores for distinct questions. The Kruskal-Wallis test was used to compare the variance of ranks in unmatched observations. Spearmans correlations were calculated to test the correlation between pain levels on the NRS, depression anxiety and stress levels and the acceptability data. P <0.05 was considered statistically significant. The sample size estimation was performed with G*Power [33]. With α = 0.05 and a power of 0.8 and an effect size of 0.25, the sample size for the Kruskal-Wallis test was estimated to be 128.

## Results

### Patient characteristics

129 patients (44 m/ 85 f) entered the study (mean age 51.5 years). Mean duration of pain was 14.7 years. Patients suffered from a variety of different pain diagnoses such as:

cervical pain,
widespread pain,
facial pain,
headache,
low back pain and
neuropathic pain, mostly of the lower limb.

A chronic pain disease with somatic and psychological factors (ICD 10: F45.41) [34] was diagnosed in 92.2% of the 129 patients. Depression was present in 43.4% and anxiety was diagnosed in 13.1% of the patients. Psychosocial factors maintaining the chronic pain disease as well as a sleep disorder was were present in more than half of the patients (57.4% and 58.1% respectively). Only 18% of the patients currently took opioids (mostly strong opioids) (Table 3).

**Table 3:** Patient demographics and characteristics.

	Mean ± (range)	Median (IQR)	n	%
Age (years)	51.5 ± 14.6 (18.0–80.9)			
Sex			44 m / 85 f	34.1 m / 65.9 f
Duration of pain/years	14.7 ± 14.5 (0.5–54)			
NRS values				
Mean pain level		7 (6–8)		
Maximal pain level		9 (8–9)		
Tolerable pain Level		2 (1–3.25)		
DASS-test				
Depression		7 (4–13)		
Anxiety		5 (2–9.25)		
Stress		10 (7–15)		
Pain diagnosis				
Headache and facial pain			31	24.0
Neck pain			12	9.3
Low back pain			42	32.6
Neuropathic Pain			20	15.5
WSP			24	18.6
Psychological diagnosis				
Chronic pain disorder (ICD10: F 45.41)			119	92.2
Depression			56	43.4
Mild			31	24.0
Medium			25	19.4
Anxiety			17	13.2
Psychosocial factors			74	57.4
Sleep disorder			75	58.1
Medication				
Strong opioids			15	11.6
Weak opioids			9	7.0

### Placebo acceptability

**Fig 1:**
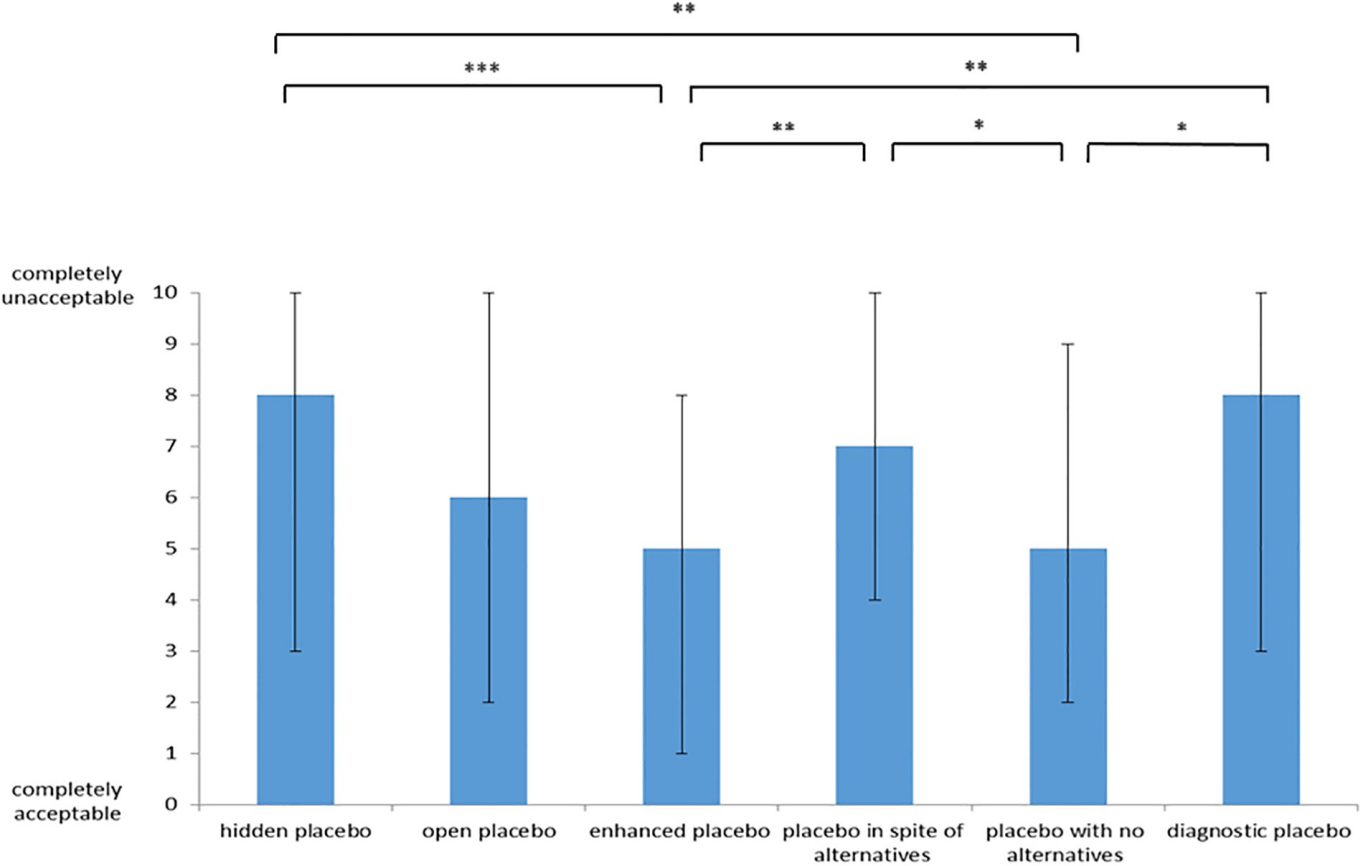
Median values of acceptability of different conditions of placebo application:, * = p< 0.05, ** = p < 0.01, *** = p< 0.001 (Friedmann Test, Post Test: Dunn`s Multiple comparison Test), error bars represent 25% and 75% percentiles.

The acceptability rate for hidden placebo use was on average rated median 8 (IQR 3–10), (mean 6.5, SD 3.7). Median acceptability values were 6 (IQR 2–10) (mean 5.6, SD 3.7) for open placebo application, 5 (IQR 1–8) (mean 4.7, SD 3.6) for enhanced placebo application, 7 (IQR 4–10) (mean 6.3 SD 3.4) for placebo use in spite of alternatives, 5 (IQR 2–9) (mean 5.2, SD 3.6) for placebo use with no alternatives and 8 (IQR 3–10) (mean 6.6, SD 3.6) for diagnostic placebo application (Fig 1). Thus, the enhanced placebo application was the only placebo condition, which was on average rated nearer to being completely acceptable then completely unacceptable.

#### Factors influencing placebo acceptability

There was no correlation between placebo acceptability and duration of pain. No difference in placebo acceptability was detected between male and female participants. No statistically significant difference was found in placebo acceptability between the different diagnosis groups. Also no statistically significant difference was found between patients with and without the diagnosis of chronic pain disease with somatic and psychological factors (ICD 10: F 45.41), with and without opioid intake, with and without sleep disorder, with and without anxiety disorder, depression or the presence of distinct psychological factors contributing to pain maintenance (Table 4). Only a moderate negative correlation of the acceptability with age of the patient was found in the diagnostic placebo condition (r = -0.3034, p = 0.0005).

**Table 4:** Placebo acceptability in different paradigms, dependent on sex, psychological diagnosis and opioid use; paradigms: Hidden = hidden placebo, open = open placebo, enhanced = enhanced placebo, alternatives = placebo in in spite of alternatives, no alternatives = placebo with no alternatives, diagnostic = diagnostic placebo, 0 = “completely acceptable”,10 = “completely unacceptable”.

paradigm:	hidden	open	enhanced	alternatives	no alternatives	diagnostic
sex						
male						
median	7.5	7.0	5.0	7.0	5.5	7.5
25% percentile	4,0	3,0	2.0	4.25	3.0	3.0
75% percentile	10.0	10.0	7.75	9.75	9.0	10.0
female						
median	8.0	5.0	4.0	7.0	4.0	8.5
25% percentile	3.0	2.0	1.0	3.0	1.0	3.25
75% percentile	10.0	10.0	9.0	10.0	9.0	10.0
p	0.6051	0.2313	0.7868	0.9837	0.2220	0.3828
psychological diagnosis						
chronic pain disorder						
median	8.0	6.0	5.0	7.0	5.0	8.0
25% percentile	3.0	2.0	1.0	4.0	2.0	3.0
75% percentile	10.0	10.0	8.0	10.0	9.0	10.0
no chronic pain disorder						
median	9.5	4.0	4.0	9.5	5.5	7.0
25% percentile	2.25	1.0	0.75	3.0	1.0	4.0
75% percentile	10.0	10.0	7.75	10.0	10.0	10.0
p	0.6472	0.7650	0.7797	0.4429	0.8404	0.8233
depression						
median	9.0	4.0	4.0	9.5	5.5	7.0
25% percentile	4.25	3.0	2.25	4.0	3.0	3.0
75% percentile	10.0	10.0	9.0	10.0	9.0	10.0
no depression						
median	7.5	5.0	4.0	6.0	4.0	8.5
25% percentile	3.0	2.0	1.0	3.0	1.0	4.0
75% percentile	10.0	10.0	8.0	10.0	9.0	10.0
p	0.3584	0.3836	0.0767	0.4491	0.1160	0.3253
anxiety						
median	5.0	5.0	4.0	5.0	4.0	8.0
25% percentile	2.0	2.0	1.0	1.0	1.5	2.5
75% percentile	10.0	9.0	8.5	8.5	8.5	10.0
no anxiety						
median	8.0	6.5	5.0	8.0	5.0	8.0
25% percentile	4.0	2.0	1.0	4.0	2.0	3.0
75% percentile	10.0	10.0	8.0	10.0	9.0	10.0
p	0.5652	0.5119	0.7575	0.0518	0.4137	0.7925
psychosocial factors						
median	8.0	6.0	5.0	7.0	5.0	8.0
25% percentile	3.0	2.0	1.0	4.0	2.0	3.0
75% percentile	10.0	10.0	8.0	10.0	9.0	10.0
no psychosocial factors						
median	7.0	6.0	4.0	7.0	5.0	9.0
25% percentile	2.25	1.0	0.75	3.0	1.0	4.0
75% percentile	10.0	10.0	7.75	10.0	10.0	10.0
p	0.3812	0.7338	0.6008	0.6402	0.7153	0.1828
continued						
sleep disorder						
median	8.0	6.0	5.0	8.0	5.0	8.0
25% percentile	3.0	2.0	1.0	4.0	2.0	3.0
75% percentile	10.0	10.0	9.0	10.0	9.25	10.0
no sleep disorder						
median	8.0	5.5	4.5	5.5	4.5	8.0
25% percentile	4.0	2.0	1.75	3.0	2.0	3.0
75% percentile	10.0	9.0	7.25	10.0	8.0	10.0
p	0.7421	0.6494	0.9654	0.2164	0.5037	0.9051
medication						
opioid use						
median	8.0	6.0	4.0	8.0	7.0	6.0
25% percentile	1.5	2.25	0.0	5.0	3.0	1.5
75% percentile	10.0	10.0	8.0	10.0	9.0	10.0
no opioid use						
median	8.0	6.0	5.0	7.0	5.0	8.0
25% percentile	4.0	2.0	1.5	3.25	2.0	3.0
75% percentile	10.0	10.0	8.5	10.0	9.0	10.0
p	0.4207	0.8493	0.4549	0.4397	0.3020	0.3282

The diagnostic placebo condition also showed a weak negative correlation with depression scores (r = -0.2282, p = 0.0105) and anxiety scores (r = -0.2234, p = 0.0123). Stress scores and the mean, maximal and tolerable NRS scores showed no correlation with placebo acceptability scores.

#### Deception, trust and negative mood in the hidden and in the open placebo condition

**Fig 2:**
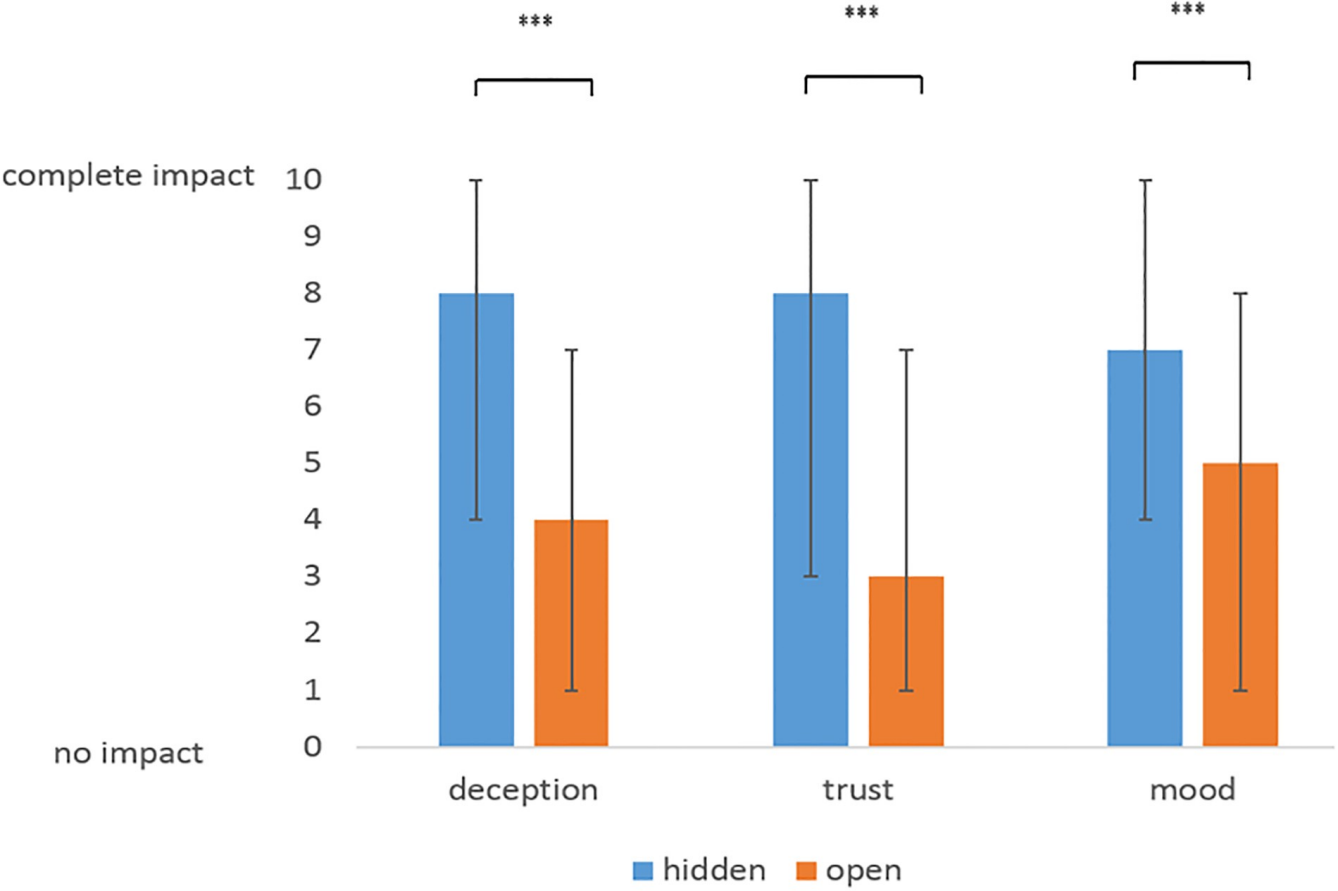
Median values of impact of placebo application on feeling of deception, trust and mood depending on way of application: Hidden and open placebo application, 0 = no impact, 10 = complete impact, *** = p < 0.0001, (Wilcoxon Matched Pairs Test), error bars represent 25% and 75% percentiles.

Overall cumulated median values for deception, trust and negative mood were much higher in the hidden condition than in the open condition (Fig 2, Table 5). There were statistically significant differences (p<0.0001) between the open and the hidden condition in every single item.

**Table 5:** Mean values for deception (0 = not deceptive, 10 = completely deceptive), trust (0 = loose no trust at all, 10 = completely loose trust) and mood (0 = no negative impact, 10 = maximal negative impact on mood).

*Paradigm*	*median*	*IQR*	*mean*	*SD*	p*	*r*
Hidden placebo—improved						
Deception	5	2–9	5.4	3.6		
Trust	6	2–10	5.6	3.8		
Mood	5	1–8	4.9	3.5		
Hidden placebo—unchanged						
Deception	9	6–10	7.7	2.8		
Trust	9	5–10	7.3	3.2		
Mood	8	5–10	7.0	3.0		
Hidden placebo—worsened						
Deception	10	7–10	8.1	2.8		
Trust	9	6–10	7.7	3.1		
Mood	9	6–10	7.8	2.8		
Hidden placebo—cumulated						
Deception	8	5–10	7.06	3.33		
Trust	8	4–10	6.89	3.50		
Mood	7	4–10	6.54	3.32		
Open placebo—improved						
Deception	3	1–5	3.5	3.3	<0.0001	0.51
Trust	2	0–6	3.2	3.3	<0.0001	0.56
Mood	3	1–5	3.4	3.2	<0.0001	0.43
Open placebo—unchanged						
Deception	4	1–8	4.6	3.6	<0.0001	0.69
Trust	3	1–8	4.3	3.7	<0.0001	0.67
Mood	5	2–8	5.0	3.4	<0.0001	0.59
Open placebo- worsened						
Deception	6	1–10	5.3	3.9	<0.0001	0.65
Trust	5	1–9	4.8	4.0	<0.0001	0.62
Mood	7	3–9	5.9	3.5	<0.0001	0.55
Open placebo—cumulated						
Deception	4	1–8	4.44	3.67	<0.0001	0.62
Trust	3	1–8	4.08	3.70	<0.0001	0.62
Mood	5	1–8	4.79	3.51	<0.0001	0.52

p*: open condition vs hidden condition (Wilcoxon matched pairs test), r: effect size.

#### Deception, trust and negative mood in the improved, constant or worsened condition

**Fig 3:**
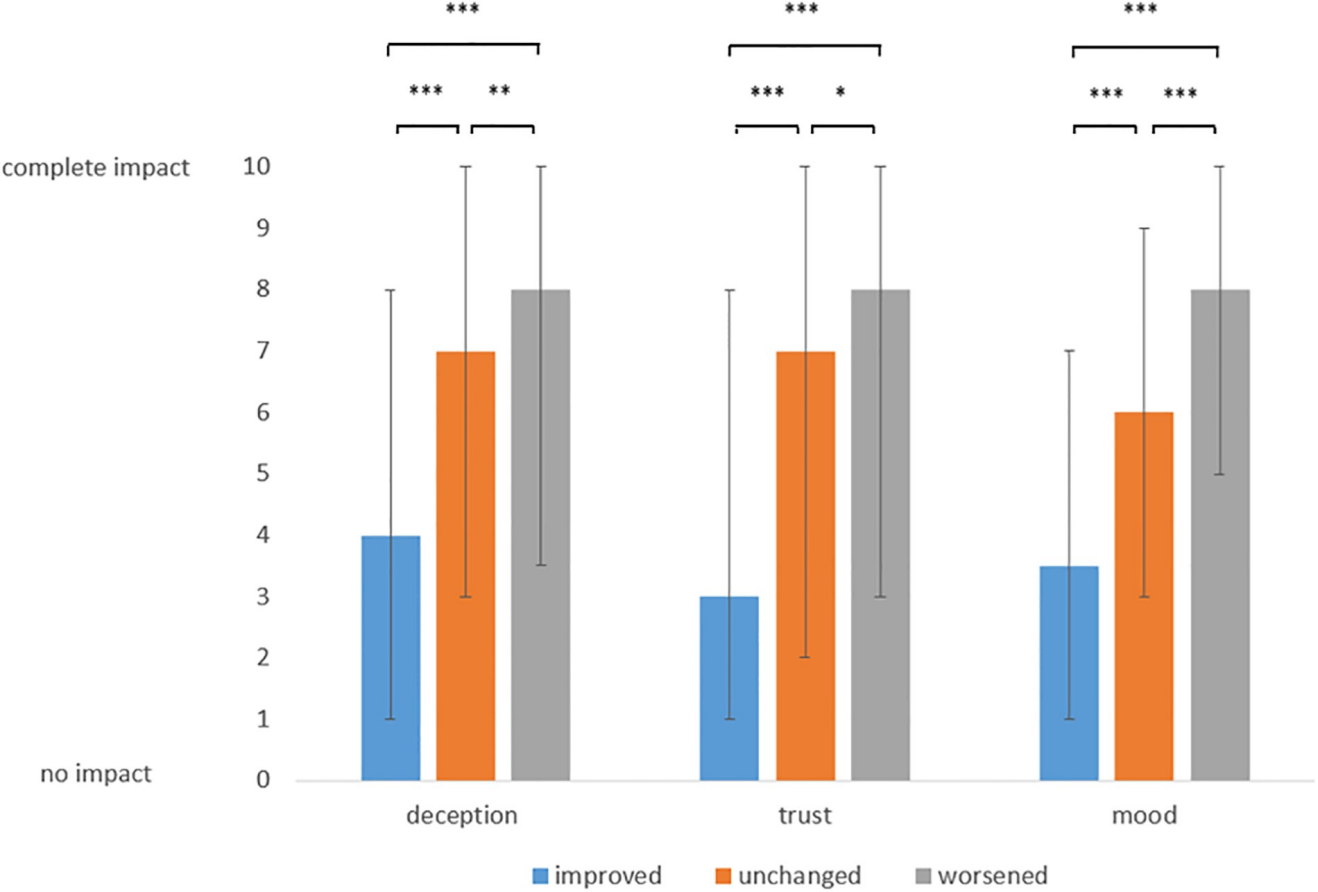
Median values of impact of placebo application on feeling of deception, trust and mood depending on change of condition: Improved, unchanged and worsened, 0 = no impact, 10 = complete impact, * = p < 0.05, ** = p < 0.01, *** = p < 0.001 (Friedman test with Dunn`s Multiple Comparison test), error bars represent 25% and 75% percentiles.

Overall cumulated median values for deception, trust and negative mood were 4 (IQR 1–8) (mean 4.3, SD 3.6) in the improved condition, 7 (IQR 3–10) (mean 6.0, SD 3.6) in the constant condition and 8 (IQR 3.5–10) (mean 6.6, SD 3.6) in the worsened condition. The difference between these values was statistically significant (p < 0.0001). The influence of the condition on deception trust and negative mood is displayed in Fig 3.

#### Factors influencing deception, trust and negative mood

There was no correlation between deception, trust and negative mood and sex or duration of pain. No statistically significant difference was found among the values for deception, trust and negative mood, between the different diagnosis groups, between patients with and without the diagnosis of chronic pain disease with somatic and psychological factors (ICD 10: F 45.41), with and without opioid intake, sleep disorder, anxiety disorder or the presence of distinct psychological factors contributing to pain maintenance. Generally, depression seemed to have some influence on the feeling of deception, impacted trust and negative mood. Here, in a number of conditions a there was a tendency towards statistical significance. Statistically significant differences between patients with and without depression were found for mood in the hidden placebo improved condition (p = 0.0499), for mood in the open placebo improved condition (p = 0.0332), and for deception in the open placebo worsened condition (p = 0.0253).

For some placebo paradigms, there were slight and moderate inverse correlations of the values for deception, impacted trust and negative mood towards age of the patient (data not shown). The condition of placebo application had a much higher net impact on deception, trust and mood (Fig 3).

Pain scores showed weak inverse correlations in the open placebo improved condition with deception (r = -0.2095, p = 0.0191), trust (r = -0.2796, p = 0.0016) and mood (r = -0.2840, p = 0.0055). Weak inverse correlations with pain scores were also seen in the open placebo unchanged paradigm with deception (r = -0.2725, p = 0.0021) and trust (r = -0.3031, p = 0.0006) and in the open placebo worsened condition with deception (r = -0.3189, p = 0.0003) and trust (r = -0.3072, p = 0.0005). The maximal pain scores and the tolerable pain scores showed no correlation with deception trust or mood.

## Discussion

The present study examined factors that might contribute to placebo acceptability by means of an adapted patient-centered systematic approach to study placebo acceptability [25–27]. In most instances, the study failed to show significant differences in placebo acceptability among patients with different pain diagnoses or accompanying psychological diagnoses or disorders. The results of the study are in extensive accordance with those of Kisaalita et al. [27]. Particularly, the rank of the individual placebo acceptability paradigms is nearly the same in both studies. For instance, in both studies hidden placebo is much more unacceptable for the patients than the enhanced placebo or the open placebo application.

Further, the change of resulting condition plays a role in placebo acceptability: an improved condition goes along with less feeling of deception, more trust and less negative mood, than an unchanged condition or a worsened condition.

The range of the values for placebo acceptability regarding hidden placebo, open placebo, enhanced placebo, placebo with or without alternatives and diagnostic placebo also reflects the findings by Kisaalita et al. [27]. While it would be common sense to expect that hidden placebo application is less acceptable than open placebo application and placebo application in spite of alternatives is less acceptable than placebo application with no alternatives, interestingly the diagnostic placebo is the least accepted placebo paradigm.

In the hidden placebo and the worsened hidden placebo paradigm, there was an inverse correlation with age for the values for deception, impacted trust and negative mood. Thus, younger patients were more likely to feel deception and react with impacted trust and negative mood than older patients.

The present study was successful in broadening the current data basis on placebo acceptability, but it widely failed in detecting specific influencing factors. No differences were detected regarding sex, pain duration, different pain diagnoses, anxiety/stress scores, psychosocial co-factors, sleep disorder or opioid use. However, older age, at least in some instances seems to go along with higher placebo acceptability in the hidden condition and in the diagnostic condition as well as less feeling of deception, impacted trust and negative mood in the hidden placebo unchanged and worsened and the open placebo worsened condition. Moreover, depression seems to increase the feeling of deception and negative mood and to decrease trust in the physician.

All in all, our data suggest that placebo acceptability is much more dependent on the context of placebo application than on factors inherent to the single patient. Up to now, apart from single physicians performing hidden placebo treatments [22], placebo use in clinical contexts has always been considered unethical and therefore has not been carried out. Therefore, the question of placebo acceptability has been a merely theoretical question. However, with growing knowledge on placebo mechanisms, open intermittent placebo applications also become conceivable [17]. Therefore, it is important to gather further knowledge about how acceptable placebo use would be for the patients under defined conditions. Our study shows that the context of its application and the resulting condition are the main predictors for the acceptance of placebo use also in severely affected chronic pain patients. As expected, patients tend to be more willing to accept an open rather than a hidden placebo application. Only the open placebo paradigm with an unchanged or improved condition is on average rated less deceptive and with less negative impact on trust and mood. The study also shows that only the enhanced placebo paradigm is on average rated as slightly more acceptable than as non-acceptable by the patients. The effectiveness of the application is also an important factor contributing to placebo acceptability. The improved condition was on average rated far more acceptable throughout all paradigms than the unchanged and the worsened condition. Interestingly, both, an open application mode as well as an improved condition seemed to have a slightly bigger positive impact on trust than on deception and mood (Tables 2 and 3).

Our data do not allow any conclusions on how or when to apply placebos in a clinical context. At best, the data might serve to give the clinician an idea of when to expect acceptance of a placebo application and when not. Anyway, due to ethical considerations, that is to say, to respect the patients`autonomy, it is crucial that the patient decides, whether to use a placebo or not. Thus, only open label applications may be considered ethical. Blease et al. [35] argue that open label placebo applications are ethical and comply with the American Medical Association (AMA) guidelines [36].

Some limitations of the study have to be discussed: participation in the study was restricted to patients undergoing multimodal pain treatment or interdisciplinary assessments. These patients were present at the hospital for at least one day. We limited the study to these patients, excluding patients coming for a single ambulatory appointment, because we sought to have time to explain to the patients that participation in the study would in no way imply a potential placebo treatment for them and that the study was only a theoretical survey, which had nothing to do with their actual treatment. This inclusion criterion however limited the number of patients eligible. Moreover, it is likely that this led to a higher portion of patients with chronic pain included in the study, as the long mean duration of pain indicates. This however was also an intended effect, as we wanted to collect data on placebo acceptability in patients with chronic pain, beyond (mild) musculoskeletal pain. The sample size estimation was difficult, as we did not have clear data in the literature on the expectable standard deviation. In any case, the sample size is sufficient to detect clinically important differences.

Another limitation is, that the study did not distinguish between open label placebo with and without rationale [13]. As patients had been informed about placebo effects in the fact sheet about the study prior to signing the agreement form patients may have answered in the sense of an open label with rationale, but this was not asked explicitly in the questionnaire.

Advantages of the study are the relatively large number of participants, the broad variation over several distinct pain diagnoses and the high participation rate.

In conclusion, the present study shows that placebo acceptability in chronic pain patients, is not, or only to a negligible degree, dependent on the pain diagnoses, pain duration and accompanying psychological factors. The paradigm of placebo application (hidden vs open, placebo with no alternatives …) and the resulting individual condition (improved, same, worse) are much stronger predictors of placebo acceptability. As the individual effect of a placebo application can not be predicted, it still difficult to decide whether or not a patient should receive an open or an open conditioned placebo. Therefore, future research might also examine the question whether placebo acceptability has an impact on the magnitude of placebo responses.

## Supporting information

S1 Table Complete set of raw data of all patients analysed in the study. (XLSX)
